# IL-17 Inhibits Oligodendrocyte Progenitor Cell Proliferation and Differentiation by Increasing K^+^ Channel Kv1.3

**DOI:** 10.3389/fncel.2021.679413

**Published:** 2021-06-22

**Authors:** Han Liu, Xueke Yang, Jing Yang, Yanpeng Yuan, Yanlin Wang, Rui Zhang, Huangui Xiong, Yuming Xu

**Affiliations:** ^1^Department of Neurology, The First Affiliated Hospital of Zhengzhou University, Zhengzhou, China; ^2^Neurophysiology Laboratory, Department of Pharmacology and Experimental Neuroscience, University of Nebraska Medical Center, Omaha, NE, United States

**Keywords:** IL-17, myelin, oligodendrocyte, Kv1.3, inflammation

## Abstract

Interleukin 17 (IL-17) is a signature cytokine of Th17 cells. IL-17 level is significantly increased in inflammatory conditions of the CNS, including but not limited to post-stroke and multiple sclerosis. IL-17 has been detected direct toxicity on oligodendrocyte (Ol) lineage cells and inhibition on oligodendrocyte progenitor cell (OPC) differentiation, and thus promotes myelin damage. The cellular mechanism of IL-17 in CNS inflammatory diseases remains obscure. Voltage-gated K^+^ (Kv) channel 1.3 is the predominant Kv channel in Ol and potentially involved in Ol function and cell cycle regulation. Kv1.3 of T cells involves in immunomodulation of inflammatory progression, but the role of Ol Kv1.3 in inflammation-related pathogenesis has not been fully investigated. We hypothesized that IL-17 induces myelin injury through Kv1.3 activation. To test the hypothesis, we studied the involvement of OPC/Ol Kv1.3 in IL-17-induced Ol/myelin injury in vitro and in vivo. Kv1.3 currents and channel expression gradually decreased during the OPC development. Application of IL-17 to OPC culture increased Kv1.3 expression, leading to a decrease of AKT activation, inhibition of proliferation and myelin basic protein reduction, which were prevented by a specific Kv1.3 blocker 5-(4-phenoxybutoxy) psoralen. IL-17-caused myelin injury was validated in LPC-induced demyelination mouse model, particularly in corpus callosum, which was also mitigated by aforementioned Kv1.3 antagonist. IL-17 altered Kv1.3 expression and resultant inhibitory effects on OPC proliferation and differentiation may by interrupting AKT phosphorylating activation. Taken together, our results suggested that IL-17 impairs remyelination and promotes myelin damage by Kv1.3-mediated Ol/myelin injury. Thus, blockade of Kv1.3 as a potential therapeutic strategy for inflammatory CNS disease may partially attribute to the direct protection on OPC proliferation and differentiation other than immunomodulation.

## Introduction

In the central nervous system (CNS), neuronal axons are myelinated with oligodendrocytes (Ols), and damage of such a myelin sheath is a striking pathological feature of white matter damage in many inflammation-related diseases, including stroke (Oksala et al., 2009), Alzheimer disease (Nasrabady et al., 2018), and in particular multiple sclerosis (MS), which is a disseminated sclerosis in CNS affecting millions of people worldwide. While the mechanisms underlying MS pathogenesis are not fully understood, it is widely accepted that myelin sheath destruction induced by autoimmune response is most likely the cause. In MS, the immune system attacks myelin sheath leading to demyelination and impairs remyelination by retardation or inhibition of the myelin-producing cells to form myelin sheath after demyelination (Boulanger and Messier, 2014). Remyelination requires Ol progenitor cell (OPC) proliferation and migration to the lesion sites, where they differentiate, eventually to mature Ols and wrap neuronal axons to form myelin sheath. Remyelination readily occurs in MS, but it is incomplete and inefficient, and the reason is unfortunately unknown. It is believed that OPC differentiation is the key step for successful remyelination based on the fact revealed by pathological studies reported that approximately 60–70% of demyelinated lesions in MS contain immature OPCs. Those immature OPCs appeared to be in an arrested state, unable to fully differentiate (Lucchinetti et al., 1999; Chang et al., 2002; Boyd et al., 2013). Although current therapies targeting at suppression of overactive immune cells, such as lymphocytes, have some promising effects in retarding disease progression (Hauser et al., 2013), there is no effective way to stimulate and promote axonal remyelination once a demyelinated lesion has occurred.

The Voltage-gated K+ (Kv) channel is a largest and rapidly growing family of ion channels. In general, Kv channels are responsible for the driving force of Ca^2+^ in non-excitable cells, thus mediating a variety of cellular activities. Previous studies have revealed an involvement of Kv channels in the regulation of Ol lineage cell proliferation and maturation (Pruss et al., 2011). It has also been shown that a decrease in Kv1.3 and Kv1.5 channel expression and outward K^+^ currents in mature Ols is essential for synthesis of myelin structural proteins, and suppression of outward K^+^ currents by increasing extracellular K^+^ concentration (e.g., 25 mM KCl) promotes Ol maturation (Dai et al., 2001). These results indicate that Kv channels play an important role in the regulation of OPC/Ol differentiation and maturation (Chittajallu et al., 2002; Tegla et al., 2011). Kv1.3 is highly elevated in memory T cells in MS brain (Rus et al., 2005) and has been reported to be an immune-regulatory target in MS and inflammatory animal models (Wang et al., 2019). The inhibition of Kv1.3 activity may attenuate CNS inflammation and thus benefit remyelination according to its crucial role in regulation of T lymphocytes (Rus et al., 2005; Schmitz et al., 2005; Eil et al., 2016; Wang et al., 2019) and microglial physiological activity (Fordyce et al., 2005; Di Lucente et al., 2018) in inflammatory brains. Thus, blockade of Kv1.3 might benefit CNS remyelination by suppression of immune response. However, Kv1.3 colocalized with OPC marker NG2 in MS brain (Tegla et al., 2011), which suggests the beneficial effects of Kv1.3 blockade on MS may not be attributable solely to immune suppression; direct protection on OPCs may also contribute to the observed outcome. Nevertheless, the role of OPC/Ol Kv1.3 on axonal remyelination in brain white matter damage is unknown. In a previous study, we showed Kv1.3 was involved in HIV Tat protein-induced Ol injury in rat corpus callosum (Liu et al., 2017a). To further evaluate the role of OPC/Ol Kv1.3 on remyelination in white matter damage, we studied the effects of interleukin 17 (IL-17) on regulation of OPC/Ol proliferation and differentiation via Kv1.3.

IL-17 is a member of cytokine family of IL-17A–F (Waisman et al., 2015). As a signature cytokine of Th17 cells, IL-17A (referred to as IL-17 hereafter) is significantly elevated in MS patients’ cerebrospinal fluid (CSF) (Kostic et al., 2014) and induces demyelination (Li et al., 2013; Waisman et al., 2015; Wang et al., 2017), due to its proinflammatory nature and direct toxicity on OPCs/Ols, as well as strong inhibitory effects on OPC maturation (Paintlia et al., 2011; Kang et al., 2013). Numerous immune-regulatory functions have been reported for IL-17 (Waisman et al., 2015; Kolbinger et al., 2016; Moser et al., 2020). It stimulates immune cell production of proinflammatory molecules leading to neurodegeneration, and its elevated levels in the CSF are associated with CNS inflammatory diseases. In addition, Kv1.3 expression has been found to correspond with an increase in IL-17 secretion in T cells (Gocke et al., 2012; Koch Hansen et al., 2014; Grishkan et al., 2015). To this end, we used IL-17 as a proinflammatory agent to investigate the inflammation-related mechanisms in OPC proliferation and differentiation through regulation of the levels of Kv1.3 expression.

## Materials and Methods

### Animals

C57BL/6 male mice (21 days) were supplied by Charles River Laboratories, Beijing, China, and were housed in groups of five at the standard conditions of 22°C ± 1°C temperature and relative humidity conditions of 55–60% in an artificially lit animal room under a 12-h period of light and dark cycle and fed water and food *ad libitum*. Only male mice were used in the present research. This study was approved by the Life Science Ethics Review Committee of Zhengzhou University.

### Human Ol Precursor Cell Culture and Differentiation

Human Ol precursor cell was introduced from ScienCell Research Laboratories (San Diego, CA). Cells were cultured in complete medium containing 89% RPMI 1640 medium (HyClone, Logan, UT) and supplemented with 10% fetal bovine serum (Biological Industries, Beit-Haemek, Israel), 100 U/mL penicillin, and 100 μg/mL streptomycin (P/S; Solarbio, Beijing, China). Cells were kept in an incubator at 37°C under a humidified atmosphere of 5% CO_2_ and 95% air. OPCs were maintained in complete medium for 24 h to adhere to the flask, after which the medium was replaced by Ol precursor cell differentiation medium (OPCDM; ScienCell Research Laboratories, San Diego, CA) to differentiate into mature Ols. The experiments using Ols were performed with cells after 6 days or otherwise indicated cultured in OPCDM. OPCDM was replaced on a daily basis.

### Demyelination Mouse Model (Two-Point Injection)

Mice were anesthetized with isoflurane (induced at 3%, and maintained at 1.2–1.6%) and positioned in a stereotaxic frame. Corpus callosum demyelination was induced by stereotaxic injection of 2 μL (1 μL for each point) of 1% lysophosphatidylcholine (LPC; Sigma, St. Louis, MO), which was an endogenous lysophospholipid that disrupts myelin-associated lipids leading to focal demyelination, in 0.9% NaCl solution at the rate of 0.5 μL/min using 1 μL microsyringe at two points of corpus callosum. Mice of sham group were injected with equal volume of saline in double-point injection: (1) left: 1.0 mm lateral to the bregma, 1.1 mm anterior, and 2.4 mm deep; (2) right: 1.0 mm lateral to the bregma, 0.6 mm anterior, and 2.1 mm deep. After injection, the needle was kept in the place for an additional 5 min to prevent backflow. The day of injection was regarded as day 0 (0 dpi). For all groups, the mice were daily administered 5-bromo-2’-deoxyuridine (BrdU, 50 mg/kg; Solarbio, Beijing, China) at 2, 3, and 4 dpi by intraperitoneal (i.p.) injection. A specific Kv1.3 blocker 5-(4-phenoxybutoxy) psoralen (PAP, 6 mg/kg; Santa Cruz Biotechnology, Santa Cruz, CA) was applied by i.p. injection for the PAP and PAP + LPC groups at 2, 3, and 4 dpi. The brain tissues were taken at 5 dpi for cryostat section or Western blot analysis.

### Electrophysiology

Isolation of Kv1.3 currents was achieved as previously described (Liu et al., 2017a). Briefly, cells were seeded onto 3.5-cm culture dishes for whole-cell voltage clamp recording. Cells were perfused with oxygenated standard bath solution contained (in mM): 140 NaCl, 5.4 KCl, 2 CaCl2, 1 MgCl_2_, 10 HEPES/NaOH, pH 7.3. The osmolarity was adjusted to 305 mOsm prior to recording by D-sucrose. The electrodes solution contained (in mM): 140 KCl, 2 CaCl2, 2 MgCl_2_, 11 EGTA, 10 HEPES/KOH, pH 7.3, and had an osmolarity of 300 mOsm. Whole-cell K^+^ currents were evoked by applying voltage steps from –150 to + 60 mV with a 15 mV increments, and current amplitudes were measured at the peak for each test potential. Current density (pA/pF) was calculated by dividing the digitized current values by whole-cell capacitance, which represents cell membrane surface area. Stock solution of Kv1.3-specific inhibitor PAP (Santa Cruz Biotechnology, Santa Cruz, CA) was prepared in deionized water. To access Kv1.3 current isolation, cells were recorded in standard bath and then superfused with PAP-contained bath solution at a concentration of 10 nM. Kv1.3-excluded currents were recorded at 20 min after perfusion. Isolated Kv1.3 currents were obtained by subtraction of Kv1.3-excluded current from total outward K^+^ currents. All experiments were done at room temperature (22–23°C). Recordings were obtained with an Axopatch-200 B amplifier (Molecular Devices, Sunnyvale, CA). Current signals were filtered at 1 kHz and digitized at 5 kHz using a Digidata 1440A interface (Molecular Devices). The current and voltage traces were displayed and recorded on a computer using pCLAMP 10.0 data acquisition and analysis software (pClamp, RRID:SCR_011323). Data were analyzed by Clampfit 10.0 (Clampfit, RRID:SCR_011323). All final graphics in the present work were constructed by GraphPad Prism 9.0 (GraphPad Prism, RRID:SCR_002798).

### 3-(4,5-Dimethylthiazol-2-yl)-2,5-Diphenyl Tetrazolium Bromide Assay

Cell proliferation was measured by 3-(4,5-dimethylthiazol-2-yl)-2,5-diphenyl tetrazolium bromide (MTT) assay. The homogeneous stable solution of 5 mg/mL was prepared by dissolving MTT (Ameresco, Solon, OH) powder in phosphate-buffered saline (PBS). Cells were seeded in a 96-well plate with complete medium. IL-17 (Absin Bioscience Inc., Shanghai, China) and AKT activator SC79 (Selleck Chemicals, Houston, TX) were added to the cell cultures for 48 h in the absence or presence of prior (30 min) added Kv1.3 antagonist PAP. Cells were then incubated with a 1:10 dilution of the MTT solution to complete medium for 3 h at 37°C. The formazan crystals converted from MTT were completely dissolved in dimethyl sulfoxide (Solarbio) for cell lysis, and the optical density (OD) was measured at 570 nm using Multiskan Spectrum (Thermo Fisher Scientific, Waltham, MA). The ratio of OD between the treated cells and the control cells reflected cell viability.

### Immunocytochemistry

OPCs were seeded on coverslips at a density of 0.02 × 10^6^/well in 24-well plates. Experimental treatments of PAP, IL-17, and SC79 were the same as described in MTT assay. After treatments as at indicated time, the prepared cells were fixed with 4% paraformaldehyde (PFA) in PBS for 20 min and punched holes in PBST supplemented with 0.1% Triton-X100 for 20 min. Cells were blocked in PBST containing 1% bovine serum albumin (BSA; Solarbio) for 30 min, and all the above experiments were performed at room temperature. Primary antibodies anti-Kv1.3 (Thermo Fisher Scientific cat. no. PA5-77618, RRID:AB_2736055) and anti-BrdU (ABclonal, cat. no.A20304, RRID:AB_2890022) were then applied to coverslips at 4°C overnight, and cells were incubated with the appropriate fluorescence-conjugated secondary antibody for 1 h at room temperature. For BrdU staining, cells on coverslips were pretreated with HCl. After washing, the coverslips were mounted in glass slides with mounting medium contained DAPI stain, and cells were visualized by a fluorescent microscope (Nikon Corporation, Tokyo, Japan). The ratio of BrdU^+^ cells to DAPI was counted, and comparisons among groups were performed.

### Immunohistochemistry

The mice injected with LPC accepted BrdU by i.p. injection at 2, 3, and 4 dpi. The mice used for cryosectioning accepted cardiac perfusion before sacrifice. After anesthesia, the mice were fixed on the operating table, and the thoracic cavity was cut to expose the heart. The left ventricle was punctured with a syringe filled with precooled saline. Then, the right auricula dextra of mice were snipped, and the syringe was pushed to allow the saline to rinse systemically through the vessels. After the effluent turned limpid, the mice were perfused with 4% PFA to systemic circulation for prefixation. Each mouse was given 200 mL saline and 200 mL PFA. The prepared brain tissue specimens were fixed in 4% PFA for 48 h, immersing in 15% and 30% sucrose for 24 h, respectively, and then embedded in optimal cutting temperature (OCT) media, freezing, and cryosectioning into 10-μm slices. The coronal brain slices were immunostained with primary antibody such as anti-myelin basic protein (MBP) antibody (Abcam cat. no. ab7349, RRID:AB_305869), anti-NG2 monoclonal antibody (Thermo Fisher Scientific cat. no. 37–2300, RRID:AB_2533306), and anti-BrdU antibody (ABclonal, cat. no.A20304, RRID:AB_2890022) to evaluate demyelination and proliferation, respectively. The sections were incubated with primary antibody followed by fluorescence-conjugated secondary antibodies for microscopic analysis. A minimum of five images were taken from each slide.

### Luxol Fast Blue Stain

Luxol fast blue (LFB) staining was used to measure the demyelination of myelin. The coronal brain slices were prepared as previously described. LFB staining was performed according to manufacturer’s instructions for the Luxol fast blue stain kit (Solarbio). The slices were immerged in LFB dye at room temperature for 12 h. The slices were rinsed with distilled water after washing off the excess dye with 95% ethanol. The slices were differentiated successively in 0.05% lithium carbonate solution and 70% ethanol. Photographs were taken with an optical microscope after sealing the slices with neutral resin.

### Western Blot Analysis

The expression levels of proteins were quantified by Western blot. MBP has three isoforms, and we examined the predominant isoform of protein band at 18.5 kDa by Western blot analysis. Following experimental treatments, cells and brain tissue were washed thrice with prechilled PBS. The whole-cell lysates were prepared in RIPA lysis buffer (Absin Bioscience Inc.) followed by clarification with centrifugation. All protein concentrations were quantified using the BCA protein assay kit (Solarbio), and 25 μg of total protein was loaded onto 10% sodium dodecyl sulfate–polyacrylamide gels, separated by electrophoresis, and transferred to a polyvinylidene difluoride (PVDF; Millipore, Bedford, MA) membrane. The PVDF membrane was then blocked in 5% non-fat dry milk in Tris-buffered saline (TBS) at room temperature for 1.5 h, followed by overnight incubation of primary antibodies diluted in 5% BSA at 4°C on the shaker. Primary antibodies were rabbit anti-Kv1.3 (Thermo Fisher Scientific cat. no. PA5-77618, RRID:AB_2736055), rat anti-MBP (Abcam cat. no. ab7349, RRID:AB_305869), rabbit anti-phospho-AKT (p-AKT; Cell Signaling Technology cat. no. 4060, RRID:AB_2315049), rabbit anti-AKT (Cell Signaling Technology cat. no. 4691, RRID:AB_915783), rabbit anti-phospho-p38 mitogen-activated protein kinase (MAPK) (p-p38; Cell Signaling Technology cat. no. 4511, RRID:AB_2139682), rabbit anti-p38 MAPK (Cell Signaling Technology cat. no. 8690, RRID:AB_10999090), and mouse anti–β-actin (Proteintech cat. no. 66009-1-lg, RRID:AB_2782959). Afterward, membranes were washed in TBS with 0.1% Tween-20 (TBST) for 10 min × three times and then incubated in diluted secondary antibody for 1 h at room temperature on a shaker. The secondary antibodies were horseradish peroxidase (HRP)–conjugated anti-rabbit (Jackson ImmunoResearch Labs cat. no. 111–035–003, RRID:AB_2313567), HRP-conjugated anti-rat (Jackson ImmunoResearch Labs cat. no. 112–035–003, RRID:AB_2338128) and HRP-conjugated anti-mouse secondary antibodies (Proteintech cat. no. SA00001–1, RRID:AB_2722565). After washing, membranes were finally incubated with ECL Western blot substrate (Solarbio) to detect HRP-conjugated secondary antibodies and imaged using the Image Lab system (Image Lab Software, RRID:SCR_014210). Band densities were measured by ImageJ software (ImageJ, RRID:SCR_003070).

### Statistical Analysis

All data are expressed as mean ± SE unless otherwise indicated. Statistical analyses were performed by one-way analysis of variance followed by a Fisher least-significant difference test for multiple comparisons. The difference between groups was considered significant at *P* < 0.05.

## Results

### Alteration of Kv1.3 Expression in Ol Lineage Cell Development

**FIGURE 1:**
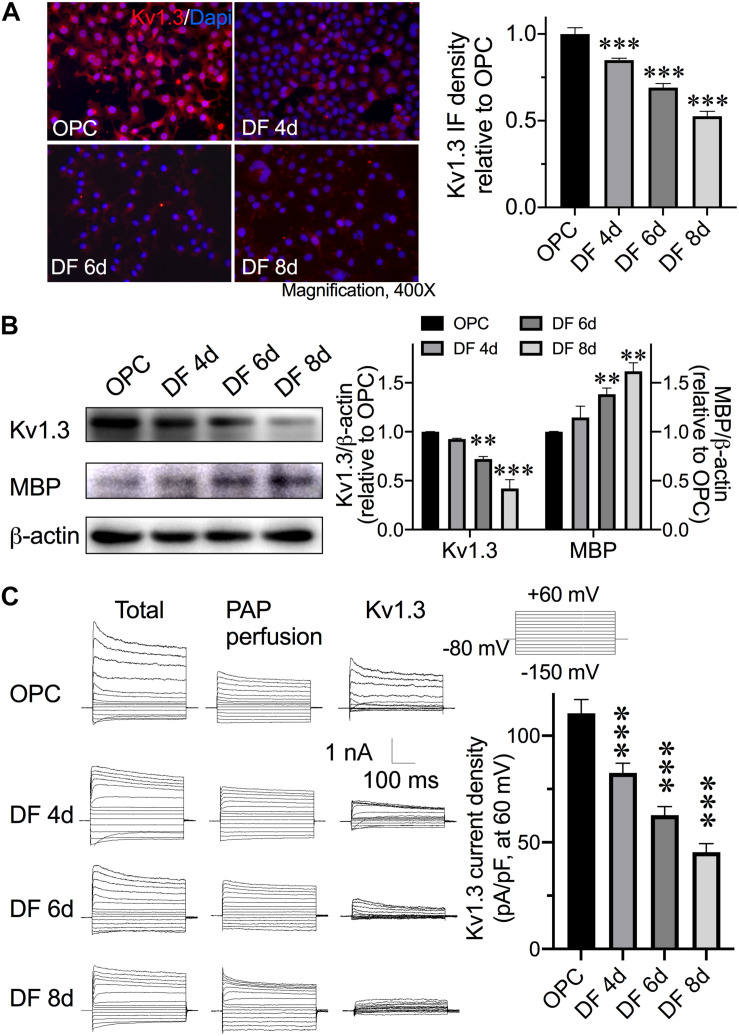
Expression of Kv1.3 in OPCs/Ols during development. OPCs were cultured in OPCDM to differentiate into mature Ols. OPCs were transferred into OPCDM for 4 days (DF 4d), 6 days (DF 6d), and 8 days (DF 8d). **(A)** Representative images of Kv1.3 immunofluorescence staining (red) in cultures. Intact cell nuclei were visualized with DAPI (blue). The immunofluorescence density of Kv1.3 was summarized in a bar graph at the right. With the maturity of OPCs, Kv1.3 decreased in a development-dependent manner. **(B)** Western blot analysis of MBP expression in cells collected from different periods of OPCs/Ols. Band densitometry data are shown in the bar graph (right). Data are normalized to β-actin shown in each gel. In contrast to the Kv1.3 alterations, the expression of MBP increased with differentiation. **(C)** Representative current traces of outward K^+^ currents recorded during depolarizing and hyperpolarizing pulses are shown in cells of OPCs, DF 4d, DF 6d, and DF 8d. The whole-cell outward K^+^ currents recorded before (Total) and 15 min after superfusion of 10 nM PAP (PAP perfusion) to the bath. The Kv1.3 currents were then isolated by subtraction of outward K^+^ currents recorded in the presence of PAP from the total currents (Kv1.3). The summary bar graph illustrating average Kv1.3 current density measured at +60 mV (pA/pF) obtained from OPCs and Ols (*n* = 16). With the maturation of Ols, the Kv1.3-conducted potassium currents decreased. All data expressed were obtained from three independent experiments unless otherwise indicated. ^∗∗^*P* < 0.01 vs. control, ^∗∗∗^*P* < 0.001 vs. control.

The expressions of MBP and Kv1.3 were first examined at different periods of development. Immunofluorescences showed the Kv1.3 protein decreased within the maturation. At the sixth day after cells were changed into OPCDM (DF 6d), the Kv1.3 immune density reduced approximately 30% of that in OPCs. At the eighth day in OPCDM (DF 8d), Kv1.3 immune density further decreased to 52.6% ± 2.8% of OPCs (Figure 1A). Similar result was observed in Western blot, the Ol Kv1.3 expression decreased approximately 28 and 58% when OPCs differentiated 6 and 8 days, respectively. In contrast to Kv1.3 expression, the MBP expression increased in the differentiating process. MBP started showing a significant increase after 6 days in OPCDM and further increased when the differentiating culture extended to 8 days (Figure 1B). MBP is a myelin structural protein that is widely used to illustrate the maturation and myelinating capability of Ols. Following the expression, we thought to test the function and activity of Kv1.3 in OPCs and Ols. Voltage clamp was performed to test the Kv1.3 currents in cultured cells. PAP 10 nM (EC_50_ = 2 nM; Schmitz et al., 2005) was perfused into the extracellular solution immediately after total currents were recorded to particularly suppress Kv1.3 currents, and then the Kv1.3-excluded currents were recorded at the same cell. The isolated Kv1.3 currents were achieved by subtraction of Kv1.3-excluded currents from total currents. Kv1.3 contributed a major component in the OPC outward K^+^ currents as shown in the first line of Figure 1C, and the current density attenuated approximately 27.9, 47.7, and 65.1% after 4 days (DF 4d), 6 days, and 8 days in OPCDM (Figure 1C). As the changes of Kv1.3 and MBP expressions both reached significant enhancement after 6 days in differentiating culture, the time point of day 6 was chosen for the following studies regarding mature Ols.

### Involvement of Kv1.3 in IL-17–Induced Inhibition of OPC Proliferation and Differentiation

**FIGURE 2:**
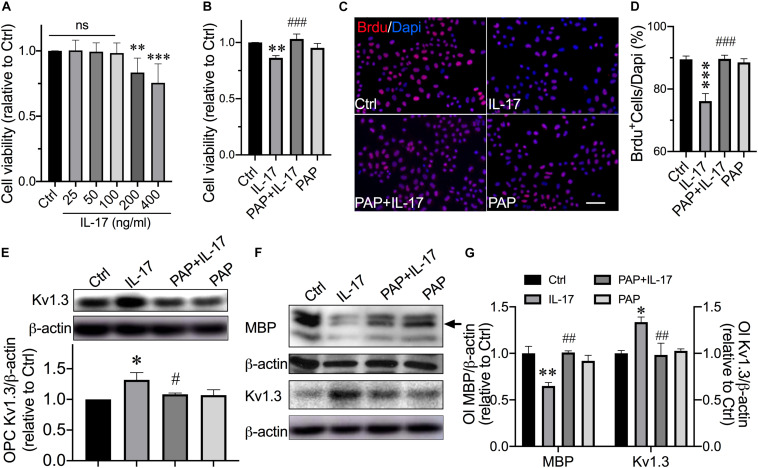
Kv1.3 blockade prevented OPCs from IL-17–induced inhibition of proliferation and differentiation. OPCs were exposed to IL-17 (200 ng/mL) for 48 h with or without preaddition of PAP (10 nM) for 30 min. **(A)** The dose of IL-17 was tittered by MTT assay performed in OPCs (*n* = 6). IL-17 significantly reduced cell viability at a concentration of 200 ng/mL and further reduced at the concentration of 400 ng/mL. **(B)** MTT assay was performed to detect OPC viability (*n* = 7). PAP pretreatment counteracted the loss of cell viability induced by IL-17. **(C)** OPCs were treated with IL-17 with or without PAP in the presence of BrdU (10 μM) for 48 h. Representative images of merged BrdU immunofluorescence staining (red) and DAPI (blue) are shown. Scale bar = 20 μm. The average percentage of BrdU^+^ cells from five independent experiments are summarized in **(D)** There were 10 randomly selected visual fields counted for each group from three independent experimental treatments. IL-17–induced reduction of BrdU^+^ cells was attenuated by PAP. **(E)** Western blot analysis of Kv1.3 expression in OPCs. Band densitometry data are shown in the bar graph (below) (*n* = 4). IL-17 treatment for 48 h elevated the Kv1.3 protein expression in OPCs, whereas the PAP attenuated this elevation. For experiments conducted with Ols in **(F,G)**, Ols were exposed to IL-17 (200 ng/mL) with or without prior addition of PAP (10 nM) for 30 min during the differentiation culture in OPCDM for 6 days. **(F**,**G)** Representative images and statistical analyses of MBP and Kv1.3 expression in mature Ols of Western blot (*n* = 3). The band marked by the arrow is a protein band with a size of 18.5 kDa. Pretreatment with PAP for 30 min counteracted the decrease in MBP and increase in Kv1.3 protein expression in Ols induced by IL-17. ^∗^*P* < 0.05 vs. control, ^∗∗^*P* < 0.01 vs. control, ^∗∗∗^*P* < 0.001 vs. control. ^#^*P* < 0.05 vs. IL-17, ^##^*P* < 0.01 vs. IL-17, ^###^*P* < 0.001 vs. IL-17.

Cytokine IL-17 level is significantly elevated in MS patients CSF, and it is found to be associated with disease severity and progression in CNS demyelination model (Kostic et al., 2014). IL-17 is demonstrated to be enhancing oxidative stress–induced Ol apoptosis (Paintlia et al., 2011) and strongly inhibiting OPC differentiation (Kang et al., 2013). Here, we tested the consequence of IL-17 on OPCs and Ols. The dose of IL-17 was tittered by MTT assay performed in OPCs. IL-17 significantly decreased OPC viability to 83.40% ± 4.5% at the concentration of 200 ng/mL and (Figure 2A, *P* = 0.0037, IL-17 200 ng/mL vs. control) further decreased to 75.5% ± 5.9% at 400 ng/mL (Figure 2A, *P* < 0.0001, IL-17 400 ng/mL vs. control). Thus, the dose of 200 ng/mL was chosen for the following studies. Moreover, IL-17 up to 400 ng/mL did not affect the OPC apoptosis (data not shown) in our culture system. Previous studies reported that Kv1.3 on T lymphocytes and microglial cells play a key role in pathophysiological processes in inflammatory brains; we next explored whether OPC/Ol Kv1.3 was directly involved in inflammation-related myelin injury. Experiments were next performed to assess the expression of OPC Kv1.3 after exposure to IL-17. Exposure of IL-17 for 48 h enhanced the Kv1.3 protein expression in OPCs (Figure 2E, *P* = 0.0114, IL-17 vs. control). PAP was preadded to culture medium 30 min prior to following addition of BrdU with or without IL-17 for 48 h. PAP mitigated IL-17–induced OPC viability reduction (Figure 2B, *P* = 0.0007, PAP + IL-17 vs. IL-17), and counteracted IL-17–caused decrease in BrdU^+^ cells percentage (Figures 2C,D; *P* < 0.0001, PAP + IL-17 vs. IL-17). These results together suggested IL-17 retarded the OPC proliferation at the concentration of 200 ng/mL, and blockade of Kv1.3 prevented IL-17–caused loss of cell viability and inhibition of proliferation. Consistent results were found in differentiated Ols. MBP is an essential myelin structural protein, which is believed to indicate the Ol myelinating ability *in vitro*. MBP classically has three isoforms due to the different transcription start sites. The analysis described below was based on the protein bands at 18.5 kDa, which is considered as the predominant isoform essential for CNS myelin stability (Smith et al., 2012; Vassall et al., 2013). IL-17 decreased the expression of MBP (Figures 2F,G; *P* = 0.0015, control vs. IL-17) and increased the Ol Kv1.3 protein expression (Figures 2F,G; *P* = 0.0106, control vs. IL-17). Pretreatment of PAP for 30 min counteracted MBP reduction (Figures 2F,G; *P* = 0.0013, PAP + IL-17 vs. IL-17) and Kv1.3 enhancement (Figures 2F,G; *P* = 0.0082, PAP + IL-17 vs. IL-17) caused by IL-17. These together suggested the involvement of Kv1.3 in IL-17–induced inhibition of OPC proliferation and differentiation.

### Kv1.3 Involved in IL-17–Induced OPC Developmental Alterations by Diminishing AKT Signal

**FIGURE 3:**
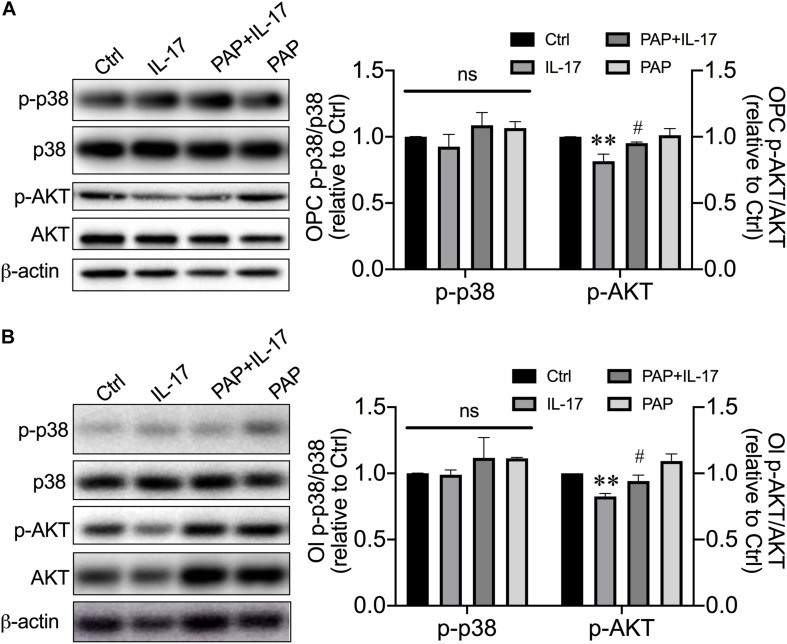
Kv1.3 involved in IL-17–induced developmental alterations by diminishing AKT signal but not p38 MAPK. OPCs were treated as described in Figure 2. **(A)** Representative images and statistical analyses of p-p38 and p-AKT expression in OPCs of Western blot (*n* = 3). IL-17 suppressed the p-AKT expression, which represented the activation level of AKT pathway in OPCs, but not p-38, whereas PAP relieved this suppression. **(B)** For experiments conducted with Ols, OPCs were transferred into OPCDM and treated the same as described in Figure 2. Representative images and band densitometry data (right) of p-p38 and p-AKT expression in Ols (*n* = 4). The attenuation of p-AKT induced by IL-17 was mitigated by PAP, indicating the integral role of Kv1.3 in IL-17–caused decline of AKT activation. ^∗∗^*P* < 0.01 vs. control, ^#^*P* < 0.05 vs. IL-17.

Thus far, we have demonstrated IL-17 up-regulated Kv1.3 protein expression in OPCs and Ols, and blockade of Kv1.3 mitigated IL-17–induced OPC proliferation and differentiation retardation. The mechanisms of Kv1.3 involvement in IL-17–caused alterations of OPC proliferation and differentiation attracted our attention. It has been shown that OPC differentiation is controlled by a number of factors, many of which act to be inhibitory including leucine-rich repeat and immunoglobulin domain-containing 1 (Mi et al., 2004, 2005), Notch-1 (Bongarzone et al., 2000; Kim et al., 2008), Wnt (Shimizu et al., 2005) etc., whereas p38 MAPK (Bhat et al., 2007; Chew et al., 2010) and AKT (Flores et al., 2008) have been shown to be required for OPC differentiation and myelination. Previous studies reported that IL-17 increased total p38 levels and restricted neural stem cell differentiating to neurons, astrocytes, and OPCs (Li et al., 2013). AKT signal is reported to be protective for myelination under IL-17–enriched conditions (Tsiperson et al., 2013; Liu et al., 2017b). We next sought to clarify whether Kv1.3 regulated OPC proliferation and differentiation through AKT and/or p38 pathways. The phosphorylation forms of p38 (p-p38) and AKT (p-AKT) proteins were examined to illustrate the activation level of these signals. IL-17 decreased the expression of p-AKT in OPCs (Figure 3A; *P* = 0.0074, IL-17 vs. control) and differentiated Ols (Figure 3B; *P* = 0.0058, IL-17 vs. control) but did not alter the activation of p-p38 in OPCs (Figure 3A; *P* = 0.94) and Ols (Figure 3B; *P* = 0.54). In both OPCs and mature Ols, PAP prevented p-AKT from IL-17–caused reduction (PAP + IL-17 vs. IL-17, *P* = 0.0297 in Figure 3A and *P* = 0.0449 in Figure 3B). These results suggested Kv1.3 may be involved in IL-17–induced inhibition of OPC proliferation and differentiation by diminishing AKT signal pathways but not p38.

**FIGURE 4:**
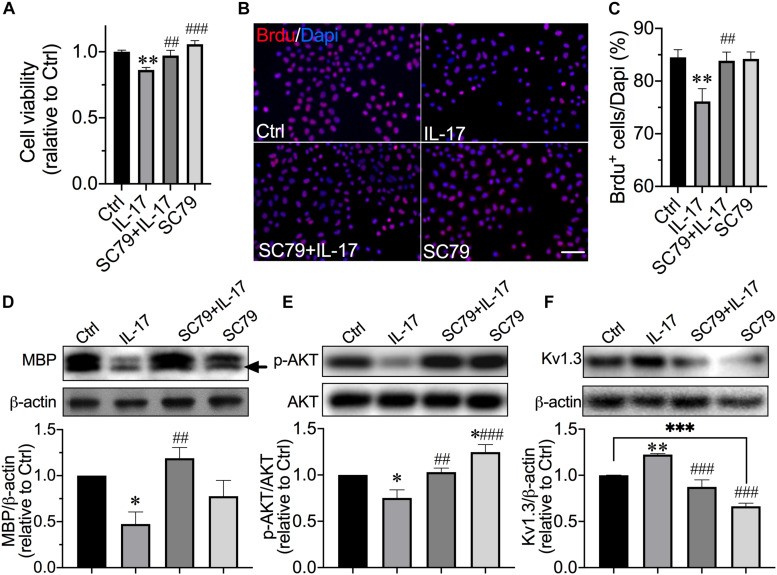
Protection of AKT activator on IL-17–induced inhibition of OPC proliferation and differentiation. OPCs were exposed to IL-17 (200 ng/mL) for 48 h with or without AKT activator SC79 (10 μM). **(A)** MTT assay was performed to detect OPC viability (*n* = 7). SC79 counteracted the decrease in cell viability induced by IL-17. **(B)** OPCs were treated as described before in the presence of BrdU (10 μM) for 48 h. Representative images of merged BrdU immunofluorescence staining (red) and DAPI (blue) are shown. Scale bar = 20 μm. The average percentage of BrdU^+^ cells from five independent experiments are summarized in **(C)** There were 10 randomly selected visual fields counted for each experimental group from three independent treatments. IL-17–induced reduction of BrdU^+^ cell percentage was attenuated by SC79. For experiments conducted with Ols in **(D–F)**, OPCs were exposed to IL-17 (200 ng/mL) with or without SC79 (10 μM) in OPCDM for 6 days. **(D–F)** Representative images and statistical analyses of MBP (*n* = 4), p-AKT (*n* = 4), and Kv1.3 (*n* = 5) expressions in Ols of Western blot. Band densitometry data are shown in the bar graph (below). SC79 effectively activated AKT signal in our culture system. Similar to the PAP, SC79 counteracted the IL-17–induced decrease in MBP and increase in Kv1.3 expression and mitigated the decrease in p-AKT induced by IL-17. ^∗^*P* < 0.05 vs. control, ^∗∗^*P* < 0.01 vs. control, ^∗∗∗^*P* < 0.001 vs. control. ^##^*P* < 0.01 vs. IL-17, ^###^*P* < 0.001 vs. IL-17.

By knowing the contribution of AKT inactivation in Kv1.3 involvement of IL-17–induced OPC cell cycle alterations, a highly selective, efficient, and cell-permeable AKT activator SC79 (Jo et al., 2012) was employed to convince the contribution of AKT. We first confirmed the efficiency of SC79 on AKT activation in our culture system after a 6-day application (Figure 4E; *P* = 0.018, SC79 vs. control). For cotreatment, SC79 was applied to culture medium at the same time with addition of IL-17. With the presence of IL-17, SC79 also performed effectively on AKT activation (Figure 4E; *P* = 0.0094, IL-17 vs. SC79 + IL-17). SC79 showed the protective effects on OPC proliferation (Figures 4A–C) and Ol MBP expression (Figure 4D). Application of SC79 protected OPCs from IL-17–induced loss of viability (Figure 4A; *P* = 0.0093, IL-17 vs. SC79 + IL-17) and mitigated IL-17–caused BrdU^+^ cell number decrease (Figures 4B,C; *P* = 0.0057, IL-17 vs. SC79 + IL-17). In differentiating culture, SC79 protected Ols from IL-17–induced attenuation of MBP expression (Figure 4D; *P* = 0.0014, IL-17 vs. SC79 + IL-17) and mitigated IL-17–caused Kv1.3 elevation (Figure 4F; *P* < 0.0001, IL-17 vs. SC79 + IL-17). These results suggested that AKT activation promotes the OPC proliferation and differentiation in exposure of IL-17, and decreased AKT activation participated in Kv1.3 involvement of IL-17–induced inhibition of OPC proliferation and differentiation.

### Kv1.3 Blockade Prevented Ols From LPC-Induced Myelin Loss *in vivo*

**FIGURE 5:**
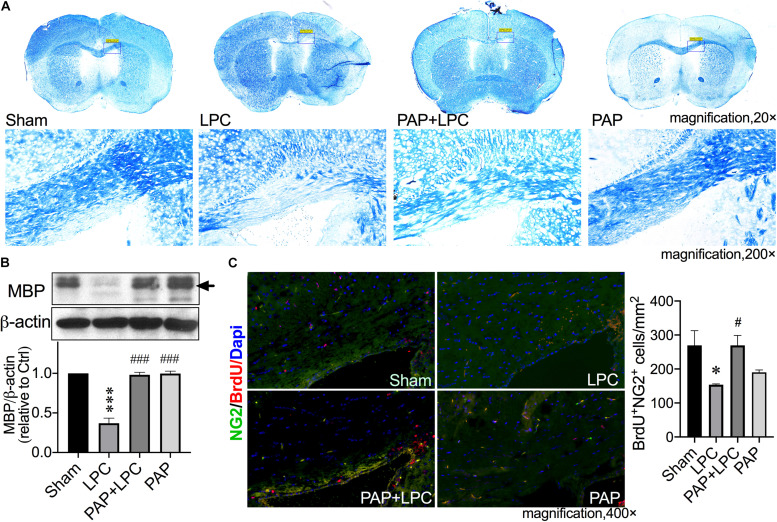
Kv1.3 blockade protected corpus callosum from LPC-induced demyelination *in vivo*. The frozen sections were obtained from the demyelination model induced by stereotaxic injection of LPC (two points, 1%, 1 μL of each point) in corpus callosum. Mice were treated with or without PAP (6 mg/kg) and accepted BrdU (50 mg/kg) by i.p. injection. **(A)** Representative images of myelin sheath LFB staining (blue) in coronal sections of animal brain tissues. The low-magnification views (first line) show the complete coronal section, in which the framed parts are enlarged into the high-magnification views showing below. Compared with sham group, the injection site of LPC reduced significantly in overall density of staining for myelin. PAP significantly improved the LPC-induced myelin damage. **(B)** Western blot analysis of MBP expression in brain tissues around the injected points dissected out from the brain. The band marked by the arrow is a protein band with a size of 18.5 kDa. Blockade of Kv1.3 significantly prevented corpus callosum from LPC-induced MBP reduction (*n* = 3). **(C)** Representative images of merged BrdU immunofluorescence staining (red), NG2 (green), and DAPI (blue) in sections. There were 10 randomly selected visual fields counted for each experimental group from three independent treatments. Cell density of BrdU^+^NG2^+^ cells are shown in the bar graph (right). The LPC-induced decrease in BrdU^+^NG2^+^ cells density was mitigated by PAP. ^∗^*P* < 0.05 vs. sham, ^∗∗∗^*P* < 0.001 vs. sham. ^#^*P* < 0.05 vs. LPC, ^###^*P* < 0.001 vs. LPC.

Having observed the MBP expression altered in cell culture, the LPC-induced demyelination mice model was utilized to interrogate myelin repair in conditions more comparable with human disease to directly disclose the effects of Kv1.3 and its blockade on myelin sheath. The LPC-induced experimental demyelination is advantageous for characterizing the focal myelin morphology because the location of the damage is known. Because corpus callosum is a sensitive marker for damage of the cerebral white matter demyelination, the areas of corpus callosum were chosen to inject LPC and observe myelination. According to the previous publication, PAP distributes in parallel in plasma and brain following enteral administration and showed even higher concentration in most tested brain regions than in plasma (Maezawa et al., 2018). The plasma concentrations following 6 mg/kg i.p. injection achieved 300 nM after 8 h and still above 10 nM after 24 h (Azam et al., 2007). Considering the PAP EC_50_ = 2 nM (Schmitz et al., 2005), a predicable concentration of greater than 10 nM is sufficient to achieve pharmacological Kv1.3 blockade in mice brains. We decided to administer PAP by i.p. injection at a dose of 6 mg/kg every 24 h. Demyelination lesions in the corpus callosum were revealed by the LFB method, which stains myelin in blue. Myelin showed intact appearance in the injection point of sham animals, while it appeared discontinuous and reduced significantly in overall density of staining in LPC-injected sites. PAP significantly improved the myelin integrity compared with that in LPC-injected corpus callosum (Figure 5A). Myelination was also evaluated by blotting reactivity of MBP. The tissue of injection areas was taken to examine MBP expression. In agreement with the morphological observation, LPC decreased the MBP expression to approximately 36% of sham (Figure 5B; *P* < 0.0001, LPC vs. sham), and this reduction was not observed in Kv1.3 inhibitor/LPC-cotreated mice (Figure 5B; *P* < 0.0001, LPC vs. PAP + LPC). By exploring the alterations of myelination, the OPC proliferation was next examined in mice. In LPC-treated mice, the number of BrdU^+^NG2^+^ cells were significantly lessened compared with sham mice (Figure 5C; *P* = 0.0147, LPC vs. sham), and administration of PAP prevented the decrease in number of BrdU^+^NG2^+^ cells (Figure 5C; *P* = 0.0147, LPC vs. PAP + LPC). These results together suggested that Kv1.3 blockade is protective for OPC proliferation and myelination *in vivo*.

## Discussion

In the present study, we demonstrated that Kv1.3 blockade effectively promoted OPC proliferation and differentiation through activation of AKT signaling, leading to protection of myelin from IL-17– and LPC- induced myelin damage *in vitro* and *in vivo*, which is beneficial to remyelination in neurological disorders with demyelination.

IL-17–induced Ol injury is closely relative to MS pathogenesis. MS is the most common cause of non-traumatic disability in young people, affecting approximately 2.5 million people worldwide (Pugliatti et al., 2002; Compston and Coles, 2008). Axonal remyelination has been a challenge in MS therapies. To date, there is no proven way to repair demyelinated lesions. Disease progression usually has two phases: relapsing–remitting phase and progressive phase. The different disease phases reflect different underlying neuropathology, with inflammation and demyelination in the relapsing–remitting phase and neurodegeneration in the progressive phase (Jolanda Munzel and Williams, 2013). Previous studies of Kv1.3 in MS and other neuroinflammatory diseases were mainly focused on the ionic immune regulating role of Kv1.3 in T cells (Rus et al., 2005; Schmitz et al., 2005; Eil et al., 2016; Wang et al., 2019) and microglia (Fordyce et al., 2005; Di Lucente et al., 2018), which were relative to the pathogenesis in relapsing–remitting phase. Here we demonstrated a direct toxic effect of IL-17–induced Kv1.3 overexpression on myelin-producing cells OPCs and Ols. Such a direct toxicity was validated by using a relatively immune-independent mouse model that maximally eliminates the probable involvement of T-cell Kv1.3 in observed results. Myelin damage causes further axonal injury related to neurodegeneration in the progressive phase, because myelinated axons appear myelin dependency during development (Alizadeh et al., 2015). Additionally, an increased number of IL-17–expressing cells were observed in ischemic brain tissues of both human and rodent (Li et al., 2005; Gelderblom et al., 2012), suggesting that IL-17 participates in the CNS pathophysiology of secondary inflammation after ischemic stroke. The MS appears to be the disease mostly associated with IL-17, although IL-17 is relatively associated with different inflammatory conditions in the CNS.

IL-17–producing subset of CD4^+^ T cells are identified as Th17 cells. The role of Th17 cells in the pathogenesis of relapsing–remitting MS has been demonstrated in several studies, showing the identification of Th17 cells in the MS lesions but not in normal-appearing white matter tissues or control brain specimens (Tzartos et al., 2008) and increased levels of Th17 cell gene expression as well as IL-17 protein in MS brain lesions (Lock et al., 2002; Komiyama et al., 2006; Tao et al., 2014). Although at certain conditions IL-17 may boost the differentiation of OPCs (Rodgers et al., 2015), most publications reported that IL-17 was harmful for OPC and Ol survival and function (Paintlia et al., 2011; Li et al., 2013; Waisman et al., 2015; Wang et al., 2017), thus promoting inflammatory injuries. Our results disclosed that IL-17 caused OPC loss of cell viability at the concentrations of 200 ng/mL or greater. Together with the results from BrdU experiments, we demonstrated that IL-17 inhibited OPC proliferation (Figure 2) without alteration of cell apoptosis (data not shown), which is consistent with previous results obtained with primary OPC culture (Rodgers et al., 2015). Similar results were also observed in neural stem cells that IL-17 stimulated neural stem cell way out of cell cycle despite no change of apoptosis (Li et al., 2013). Controversial results were reported in primary OPCs cultured with IL-17 for 4 days at the concentrations of 50 and 200 ng/mL (Kang et al., 2013). We next examined the effect of IL-17 on OPC differentiation. Application of IL-17 into OPCDM for 6 days at a concentration of 200 ng/mL inhibited the OPC differentiation (Figure 2). Kang et al. claimed an inhibitory effect on OPC maturation of 25 ng/mL IL-17 addition to culture medium with absence of proliferative signal platelet-derived growth factor (PDGF) (Kang et al., 2013). Rodgers et al. observed an enhancement of OPC differentiation in both absence and presence of PDGF, while culture treated with IL-17 at concentrations of 25 and 50 ng/mL within the first 2 h of plating (Rodgers et al., 2015). These contrast findings suggest the role of IL-17 on OPC differentiation is highly time- and dose-dependent. The discrepancies may also attribute to the culture system and stage of cells within the Ol lineage.

Kv1.3 is highly expressed in postmortem MS brain plaques, localized on inflammatory infiltrates (Rus et al., 2005) and OPCs (Tegla et al., 2011). These findings indicate the involvement of Kv1.3 in MS pathogenesis. Indeed, several groups reported the contribution of Kv1.3 in MS-related pathogenesis, and most data were from inflammatory cells in experimental autoimmune encephalomyelitis (EAE) model. Blockade of Kv1.3 revealed beneficial effects on alleviation of EAE (Huang et al., 2017; Fan et al., 2018; Yuan et al., 2018; Zhao et al., 2020). Preclinical trials targeting at Kv1.3 to treat autoimmune disease, including neuroinflammatory MS, were conducted and showed positive results (Perez-Verdaguer et al., 2016; Prentis et al., 2018). Although it is known that Kv1.3 is expressed on OPCs in MS lesion, the role of OPC/Ol Kv1.3 in MS pathogenesis is less appreciated. We showed that Kv1.3 currents and channel expression decreased during the OPC development in the cell culture (Figure 1), which is in agreement with previous observation on primary cultures (Chittajallu et al., 2002). IL-17–induced reduction of OPC proliferation and MBP expression in cultures were prevented by Kv1.3 antagonist, but antagonist alone did not alter proliferation and MBP expression (Figure 2), suggesting that Kv1.3 is involved in MS-related myelin damage, and Kv1.3 blockade affects OPC cell cycle particularly under pathological conditions. This may explain the controversial results from Vautier et al. (2004) that overexpression of Kv1.3 enhanced OPC proliferation but did not interfere differentiation when conducted under relatively physical conditions. Although we demonstrated an enhancement of Kv1.3 expression after IL-17 treatment, the molecular mechanisms remain obscure. In microglial cells, Kv1.3 expression is elevated by ERK1/2 activation (Liu et al., 2013), which is downstream molecular of IL-17. IL-17 may increase Kv1.3 expression potentially by activating ERK1/2 signal. AKT (Flores et al., 2008) and p38 MAPK (Bhat et al., 2007; Chew et al., 2010) are the main positive signals for OPC development. It is reported that AKT phosphorylation is dependent on Kv1.3 activation in Ol lineage cells and situated downstream of Kv1.3 (Tegla et al., 2011), and coexpression of PKB/AKT with Kv1.3 in oocytes enhanced the Kv1.3 channel abundance, suggesting AKT up-regulates Kv1.3 expression (Warsi et al., 2015). In both natural killer cells and T cells, Kv1.3 seems to activate AKT/mTOR signal cascade (Eil et al., 2016; Geng et al., 2020). Together, these findings suggest that Kv1.3 and AKT act synergistically. We examined the involvement of p38 MAPK and AKT in Kv1.3-mediated reduction of OPC proliferation and differentiation. The activation of AKT but not p38 MAPK was inhibited by IL-17–induced Kv1.3 enhancement, which was counteracted by AKT activator SC79 (Figures 3, 4). This is a novel pattern different from previously demonstrated synergism of Kv1.3 and AKT. It is worth pointing out that downstream molecules of IL-17, including but not limited to ERK (Amatya et al., 2017; Xie et al., 2020; Allan et al., 2021), JNK (Amatya et al., 2017; Milovanovic et al., 2020), and nuclear factor κB (Jiang et al., 2017; Wu et al., 2018), may also contribute to the observed alterations of OPC cell cycle caused by IL-17, which were not examined in the present study. In addition, studying the role of the aforementioned negative signals in OPC development may be needed in the future, which will help us better understand the regulatory complex.

EAE is the most utilized animal model to explore MS-related pathogenesis and therapeutic strategy. The current immunosuppressive therapies are mainly based on the research done with EAE. Indeed, EAE is very heterogeneous regarding induction methods, clinical and pathological features, and amenability to treatments. The idea of EAE is based on the hypothesis that MS is mediated by autoreactive T-cell infiltration into CNS. But this hypothesis is challenged by studies that demonstrate occurrence of Ol death as the very early and perhaps the initial event in the pathology of the plaque, even before development of inflammation (Barnett and Prineas, 2004; Trapp, 2004). This brings EAE limitations when applied to human disease (Sriram and Steiner, 2005; Gold et al., 2006), particularly for remyelination. However, we focused on the OPC/Ol Kv1.3 function in inflammatory brains; LPC-induced demyelination was chosen to minimize the role of infiltrated T-cell Kv1.3 in the present study. Each model has its advantages and limitations. Other elements cannot be fully eliminated in LPC model, for example, the resident microglia around lesion site (Chen et al., 2020), which also expresses Kv1.3. Global administration of PAP may have the potential to inhibit Kv1.5 activity. However, the selectivity of PAP for Kv1.3 is approximately 23-fold higher than Kv1.5 (Schmitz et al., 2005; Vicente et al., 2006), making the possibility of Kv1.5 activation ignorable. In addition, systemic application of PAP to animals may also target on neuronal and microglial Kv1.3 channels; such a potential might be minimal as only small amounts of Kv1.3 are expressed on neurons in some brain regions.

In summary, our current data showed that IL-17 induced inhibition of OPC proliferation and differentiation through Kv1.3 channel, resulting in retardation in axonal remyelination and consequent brain white matter damage. We, for the first time, demonstrated that the integral role of Kv1.3 in IL-17 caused ATK signal decline, resulting in inhibitory effects of OPC proliferation and differentiation. Collectively, these findings serve to define the pathogenesis of CNS inflammation–induced myelin damage and support a potential therapeutic strategy of using Kv1.3 blocker to treat MS and white matter damage in other neurological disorders.

## Data Availability Statement

The raw data supporting the conclusions of this article will be made available by the authors, without undue reservation.

## Ethics Statement

The animal study was reviewed and approved by the Life Science Ethics Review Committee of Zhengzhou University.

## Author Contributions

HL conceived and designed the project and carried out experiments together with XY. YW and YY participated in collating the experimental date and analysis. HL, XY, JY, and RZ edited the manuscript. HX and YX contributed to the revision of the manuscript and gave pertinent opinions. All authors contributed to the article and approved the submitted and final published version.

## Conflict of Interest

The authors declare that the research was conducted in the absence of any commercial or financial relationships that could be construed as a potential conflict of interest.
